# Comprehensive analysis of *Eleutherococcus senticosus* (Rupr. & Maxim.) Maxim. fruits based on UPLC–MS/MS and GC–MS: A rapid qualitative analysis

**DOI:** 10.1002/fsn3.3887

**Published:** 2023-12-13

**Authors:** Yaodan Chang, Yong Jiang, Jingnan Chen, Sen Li, Yimeng Wang, Linlin Chai, Jingwen Ma, Zhibin Wang

**Affiliations:** ^1^ Key Laboratory of Basic and Application Research of Beiyao, Ministry of Education Heilongjiang University of Chinese Medicine Harbin China; ^2^ Department of Rheumatism, The First Affiliated Hospital Heilongjiang University of Chinese Medicine Harbin China; ^3^ Department of Acupuncture, The Second Affiliated Hospital Heilongjiang University of Chinese Medicine Harbin China

**Keywords:** antioxidant activity, *Eleutherococcus senticosus* (Rupr. & Maxim.) Maxim., GC–MS, UPLC–QTOF–MS/MS

## Abstract

*Eleutherococcus senticosus* (Rupr. & Maxim.) Maxim. fruits (ESF), as a natural edible fruit, has long been popularized. However, few studies have conducted comprehensive chemical analyses of it. This study aimed to assess nonvolatile, volatile, and fatty oil components of ESF and to preliminarily explore the antioxidant activities. The qualitative and quantitative analyses of volatile and fatty oil components of ESF from 15 different regions were performed by the gas chromatography–mass spectrometry (GC–MS). Totally, 37 and 28 compounds were identified from volatile oil and fatty oil, respectively. The ultra‐high‐performance liquid chromatography–quadrupole time‐of‐flight mass spectrometry (UPLC–QTOF–MS/MS) was used to accurately detect 43 compounds of nonvolatile components. The volatile and fatty oil components and nonvolatile components of ESF were used as samples to determine the antioxidant activity of 2,2‐diphenyl‐1‐picrylhydrazyl (DPPH) in vitro. The components of ESF had antioxidant activity, and the nonvolatile components had stronger antioxidant activity. The results revealed that the proposed method, which is of great significance for the screening of new active ingredients, is valuable for the identification of pharmaceutical component and further development of food industry.

## Graphical abstract

UPLC–QTOF–MS/MS and UNIFI were used to identify 43 nonvolatile components of ESF. GC–MS and NIST14.L identified 65 volatile components in ESF from different areas. The antioxidant effects of ESF volatile components in vitro were investigated.

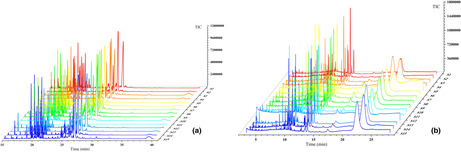

## 1 INTRODUCTION

*Eleutherococcus senticosus* (Rupr. & Maxim.) Maxim. (ES), also known as *Acanthopanax senticosus* and Siberian ginseng, is a perennial herb of the Araliaceae family that is mainly distributed in Russia, China, Korea, and Japan, especially in Heilongjiang, Jilin, and Liaoning provinces in the northeast China (Jia et al., 2021). According to the Chinese Pharmacopoeia, ES can invigorate qi and strengthen spleen, tonify kidney, and calm the mind (Committee, 2020). In the European Union, ES has been used for more than 40 years, the European Medicines Agency listed ES root as an herb that can treat symptoms such as fatigue in 2014, and in the 14th edition of the Russian Pharmacopoeia, there is also a monograph on ES (Shikov et al., 2021). Modern pharmacological studies have shown that various components of ES have been widely used in traditional medicine, including root, bark, leaf, and fruit, and they have several pharmacological activities, such as antioxidant, anti‐inflammatory, and treatment of neurodegenerative diseases (Jiang & Wang, 2015; Kim et al., 2020; Xia et al., 2020; Zhou et al., 2023). And most importantly, ES is an adaptogen, and the extract of this natural plant acts as an adaptogen to improve the adaptability, resilience, and survival rate of organisms to stress (Gerontakos et al., 2020; Panossian et al., 2021).

As a type of delicious renewable berry, the fruit of ES (ESF) can be soaked in water, and it can also be made into fruit wine, fruit vinegar, and other products, playing an important role in daily health care (Liu, 2019). To date, chemical studies on ESF have mainly concentrated on its isolation and identification, and it has been proved that ESF typically contains terpenoid, flavonoid, and phenylpropyl compounds, as well as polysaccharides. Recent experiments have isolated new oleanane‐type triterpenoid saponins and sesquiterpenoids from ESF, and demonstrated that these new compounds have certain activities in cells (Zhang et al., 2021). In addition, a comprehensive and in‐depth mass spectrometry analysis and cleavage regularity of triterpenoid saponins in ESF have been conducted, proving that triterpenoid saponins in ESF can significantly reduce the damage of β‐amyloid‐induced neural network and play a neuroprotective role (Zhou et al., 2023). Bioactive compounds have also been found in rat sera after ESF administration (Han et al., 2017). Eleutherosides E and B are important compounds in ESF, which can increase the secretion of IL‐10 and thus reduce viral replication in VSV PBLs‐Int model. These studies have demonstrated that the compounds in ESF have immunostimulatory activities (Gerontakos et al., 2021; Graczyk et al., 2021). A previous study found that the antioxidant activity of ESF could be related to the contents of polysaccharides (Zhao et al., 2013). However, there is no comprehensive analysis of the nonvolatile, volatile, and fatty oil components of ESF and their antioxidant activities; thus, it is essential to further explore such components. The gas chromatography–mass spectrometry (GC–MS) can analyze volatile components, label compounds quantitatively, and combine with stoichiometric methods to distinguish plants growing in different regions (Bai et al., 2021). The ultra‐high‐performance liquid chromatography–quadrupole time‐of‐flight mass spectrometry (UPLC–QTOF–MS/MS) is an efficient technique in the chromatographic separation, and it has been successfully employed for its fast, high‐resolution separation with the satisfactory sensitivity. GC–MS and UPLC–MS/MS technologies have been widely used for the separation and rapid identification of compounds in natural plants (Liu et al., 2022; Pan et al., 2019).

In order to analyze and evaluate the volatile and fatty oil components and quality of ESF from different production areas and assess the composition and structural cracking principle of nonvolatile compounds, a new rapid and sensitive UPLC–MS/MS method for the detection of major or trace components was, for the first time, proposed in this study. In addition, GC–MS was used to obtain fingerprints and relative area percentage from different origin of ESF volatile and fatty oil components. This qualitative and quantitative methods based on UPLC–MS/MS and GC–MS can be utilized for the quality assessment of ESF. Hence, this study may provide a reliable basis for ESF to a certain extent and for its further rational development and utilization.

## 2 MATERIALS AND METHODS

### 2.1 Materials

Totally, 15 batches of dried ESF were collected from different regions from August to October 2022, which were mainly produced in Heilongjiang, Jilin, and Liaoning provinces in China (Table 1, Figure S1). After picking the ripe fruits, wash them in tap water and ultrapure water to remove impurities, and then dry them in a cool place. They were identified as dried fruits of ES by Professor Zhenyue Wang from the School of Pharmacy, Chinese Medicine Resource Center, Heilongjiang University of Chinese Medicine (Harbin, China).

**TABLE 1:** Distribution of different regions related to ESF extraction.

No.	Region	No.	Region
S1	Harbin, Heilongjiang Province	S9	Yanji City, Jilin Province
S2	Antu County, Yanbian Korean Autonomous Region, Jilin Province	S10	Siping City, Jilin Province
S3	Chibei District, Baishan City, Jilin Province	S11	Shuangyashan City, Heilongjiang Province
S4	Benxi City, Liaoning Province	S12	Shangzhi City, Heilongjiang Province
S5	Dunhua City, Jilin Province	S13	Wudalianchi City, Heilongjiang Province
S6	Xunke County, Heihe City, Heilongjiang Province	S14	Huadian City, Jilin Province
S7	Hulunbuir City, Inner Mongolia autonomous Region	S15	Yichun City, Heilongjiang Province
S8	Tonghua City, Jilin Province		

### 2.2 Instruments and reagents

MS spectra were acquired using a Synapt G2‐SI Accurate‐Mass Q‐TOF instrument (Waters Corp., Milford, MA) and a 7890A‐5975C system (Agilent Technologies, Inc., Santa Clara, CA). An ACQUITY UPLC HSS T3 column (1.8 μm, 2.1 × 100 mm, Waters Corp.) was used to perform LC–MS analysis; a DB‐1701 GC–MS column (30 m × 250 μm × 0.25 μm, Agilent Technologies, Inc.) was utilized to carry out GC–MS analysis. LC–MS grade acetonitrile and formic acid were purchased from Thermo Fisher Scientific (Waltham, MA, USA). N‐hexane, potassium hydroxide, methanol (LC grade), and anhydrous sodium sulfate were all purchased from Xilong Scientific Co., Ltd. (Silong, China). Water required for UPLC was purified by a Milli‐Q water purification system (Darmstadt, Germany); DPPH was purchased from Shanghai Yuan Ye Bio‐Technology Co., Ltd. (Shanghai, China).

### 2.3 GC–MS analysis

The HP‐5 MS elastic quartz capillary column (30 m × 250 μm × 0.25 μm) was utilized for GC–MS analysis. In the programmed temperature condition, the temperature of volatile oil increased from 50 to 250°C at 5°C/min. The temperature of fatty oil was kept at 80°C for 1 min, then it was heated from 80 to 250°C at 10°C/min, and was kept at 250°C for 10 min. The running time of volatile oil was 40 min and that of fatty oil was 28 min. The temperature of the injector used was 250°C, the carrier gas was high purity helium (99.999%), and the flow rate was 3.0 mL/min. The column pressure was 9.785 psi, the solvent delay time was 6 min, the sample size was 1 μL, and the injector operated was in split mode, with a ratio of 40:1. The ion source was EI ion source, the electron energy was 70 eV, and the mass range was *m*/*z* 50 ~ 550. The temperatures of ion source, transmission line, and quadrupole were 230°C, 280°C, and 150°C, respectively. The mass spectrum retrieval standard library was NIST14.L standard spectrum library.

### 2.4 UPLC–MS/MS analysis

An ACQUITY UPLC system (Waters Corp.) in tandem with a QTOF Synapt G2‐SI mass spectrometer (Waters Corp.) was utilized for qualitative analysis using an ACQUITY UPLC HSS T3 column (1.8 μm, 2.1 × 100 mm, Waters Corp.). The chromatographic separation was carried out at an ambient temperature of 35°C. The gradient of the eluent mobile phase included acetonitrile with 0.1% formic acid (A) and water with 0.1% formic acid (B) as follows: 0–1 min, 2% A; 1–3 min, 2%–10% A; 3–5 min, 10%–20% A;5–9 min, 20%–55% A; 9–13 min, 55%–70% A; 13–19 min,70%–80% A; 19–22 min, 80%–98% A; 22–22.5 min, 98%–2%A; and 22.5–23 min, 2% A. The flow rate was set at 0.2 mL/min, with a 1‐μL injection volume. The MS parameters were optimized as follows: scan type: positive and negative, acquire Mse over the range of 100–1300 Da; scan time: 0.25 s, collision energy: 20–35 V, cone voltage: 40 V.

### 2.5 Preparation of sample solutions

#### 2.5.1 Extraction of volatile and fatty oil from ESF

##### Extraction of volatile oil

Volatile oil was obtained from ESF (200.2 g) by reflux condensation for 5 h, according to the Chinese Pharmacopeia 2020 (Committee, 2020). Volatile oil was dried over Na_2_SO_4_, centrifuged at 13,000 rpm for 10 min, and stored at 4°C until further analysis. Following the same procedure, all 15 components of ESF were acquired.

##### Extraction and methyl esterification of fatty oil

ESF (30.04 g) was weighed and 450 mL *n*‐hexane was added at the ratio of 1:15 (M/V). Under the condition of ultrasonic power of 250 W, ultrasonic extraction was carried out for 30 min. After vacuum filtration, the fatty oil was obtained by rotating evaporation in water bath (60°C) until no n‐hexane was emitted. Then, 4 mL of 0.6 mol/L potassium hydroxide solution, methanol, and n‐hexane were added, respectively. After the mixture was evenly mixed and bathed at 60°C for 30 min, 10 mL distilled water was added and stratified. The upper layer was dried with Na_2_SO_4_, centrifuged at 13,000 rpm for 10 min, and stored at 4°C for further analysis. Following the same procedure, all 15 components of ESF were acquired.

##### Extraction of nonvolatile compounds from ESF

ESF (20.02 g) was randomly weighed, 30 mL of 70% methanol at a ratio of 1:15 (M/V) was added, stirred and mixed, ultrasonically extracted for 1 h, leached at room temperature, and centrifuged at 12,000 rpm for 10 min, in which the supernatant was the aqueous extract of ESF.

### 2.6 Determination of antioxidant activities of volatile components using DPPH assay

With consideration of vitamin E as the control group, 100.3 mg of volatile oil and fatty oil produced in S1–S15 were taken, and dimethyl sulfoxide (DMSO) was added to obtain 10.03 mg/mL sample solution, which was diluted to 8.031, 4.022, 1.982, 0.5021, 0.2506 mg/mL, respectively. Then, 150 μmol/L DPPH solution was prepared, sample solution (100 μL) and DPPH solution (100 μL) were added to the 96‐well plate, mixed and reacted at room temperature for 30 min in the dark, and the absorbance was measured at 517 nm. The percentage of DPPH inhibition was calculated as follows:

$$\text{Percentage of DPPH inhibition}=\left(1-\left[A_{\text{sample}}-A_{\text{control}}\right]/A_{\text{blank}}\right)\times 100\%.$$

### 2.7 Determination of antioxidant activities of nonvolatile components using DPPH assay

The solution, as prepared at Section 2.5.1, was taken and diluted with 70% methanol successively to produce 70% methanol solution with the concentrations of 0.3131, 0.6252, 1.252, 2.503, 5.021, and 10.03 g L^−1^, and the other steps were the same as those of Section 2.6.

## 3 RESULTS AND DISCUSSION

### 3.1 The chromatograms of the nonvolatile, volatile, and fatty oil components

#### 3.1.1 Volatile and fatty oil components of ESF

**FIGURE 1:**
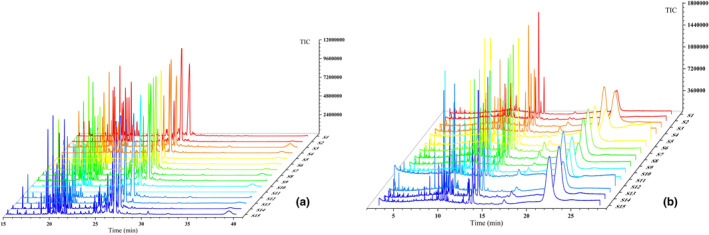
TIC chromatograms of volatile ESF components (top) and fatty ESF components (bottom) from different regions (S1–S15).

Total ion chromatograms of volatile oil and fatty oil of 15 batches of ESF were collected under optimized chromatographic conditions (Figure 1, Tables 2 and 3). By comparing the GC–MS retention time of 15 chromatograms, the obtained mass spectra were matched with the standard mass spectra in the NIST14.L library and the literature. Notably, 37 and 28 compounds were identified in volatile oil and fatty oil, respectively.

**TABLE 2:** Qualitative analysis results of volatile ESF components.

Peak number	*t*_*R*_ (min)	Chemical Abstract Service (CAS) number	Formula	Compounds
1	16.284	20307‐84‐0	C_15_H_24_	*δ*‐Elemene
2	17.213	3856‐25‐5	C_15_H_24_	(−)‐*α*‐Copaene
3	17.23	21391‐98‐0	C_10_H_16_O	Phellandral
4	18.047	515‐13‐9	C_15_H_24_	*β*‐Elemene
5	18.851	87‐44‐5	C_15_H_24_	(−)‐*β*‐Caryophyllene
6	19.634	18794‐84‐8	C_15_H_24_	(E)‐*β*‐Farnesene
7	19.673	3228‐02‐2	C_10_H_14_O	4‐Isopropyl‐3‐methylphenol
8	19.679	499‐75‐2	C_10_H_14_O	Carvacrol
9	19.815	6753‐98‐6	C_15_H_24_	(±)‐*α*‐Humulene
10	20.07	30021‐74‐0	C_15_H_24_	γ‐Muurolene
11	20.416	5951‐61‐1	C_15_H_24_	Naphthalene
12	20.535	17699‐05‐7	C_15_H_24_	*α*‐*Trans*‐Bergamotene
13	20.575	23986‐74‐5	C_15_H_24_	Germacrene D
14	20.58	13744‐15‐5	C_15_H_24_	*β*‐Cubebene
15	20.694	473‐13‐2	C_15_H_24_	*α*‐Selinene
16	20.801	495‐61‐4	C_15_H_24_	*β*‐Bisabolene
17	20.898	1461‐03‐6	C_15_H_24_	*β*‐Himachalene
18	20.96	502–61‐4	C_15_H_24_	(E,E)‐*α*‐Farnesene
19	21.091	483‐75‐0	C_15_H_24_	1‐Isopropyl‐4,7‐diméthyl‐1,2,4a,5,6,8a‐hexahydronaphtalène
20	21.096	39029‐41‐9	C_15_H_24_	(1*R*,4a*S*,8a*S*)‐1‐Isopropyl‐7‐methyl‐4‐methylen‐1,2,3,4,4a,5,6,8a‐octahydronaphthalin
21	21.198	483‐76‐1	C_15_H_24_	(+)‐*δ*‐Cadinene
22	21.431	20307‐83‐9	C_15_H_24_	*β*‐Sesquiphellandrene
23	21.589	29837‐07‐8	C_15_H_24_	Cyclohexene
24	22.462	3242‐08‐8	C_15_H_24_	Elixene
25	23.137	23262‐34‐2	C_15_H_22_O	Dendrolasin
26	23.636	25246‐27‐9	C_15_H_24_	(−)‐Alloaromadendrene
27	24.554	6750‐60‐3	C_15_H_24_O	Espatulenol
28	24.656	1139‐30‐6	C_15_H_24_O	(−)‐*β*‐Caryophyllene epoxide
29	24.832	130930‐56‐2	C_12_H_18_	Bicyclo[2.2.1]hept‐2‐ene, 2‐ethenyl‐1,7,7‐trimethyl‐
30	25.291	42558‐37‐2	C_10_H_18_	Bicyclo[3.3.1]nonane, 2‐methyl‐, (1*R*,2*S*,5*R*)‐rel‐
31	25.297	19888‐34‐7	C_15_H_24_O	(−)‐Humulene epoxide II
32	25.523	88‐84‐6	C_15_H_24_	Guaiene
33	25.818	22567‐36‐8	C_15_H_26_O_2_	(−)‐*α*‐Bisabolol
34	25.886	26184‐88‐3	C_15_H_26_O_2_	Bisabolol oxide B
35	26.068	67517‐14‐0	C_15_H_22_	2H‐2,4a‐Methanonaphthalene, 3,4,7,8‐tetrahydro‐8,8,9,9‐tetramethyl‐
36	26.544	4630‐07‐3	C_15_H_24_	3‐Isopropenyl‐4a,5‐dimethyl‐1,2,3,4,4a,5,6,7‐octahydronaphthalene
37	26.912	515‐69‐5	C_15_H_26_O	*α*‐Bisabolol

**TABLE 3:** Qualitative analysis results of fatty ESF components.

Peak number	*t*_R_ (min)	Chemical Abstract Service (CAS) number	Formula	Compounds
1	3.769	611‐14‐3	C_9_H_12_	2‐Ethyltoluene
2	4.175	526‐73‐8	C_9_H_12_	1,2,3‐Trimethylbenzene
3	4.604	95‐63‐6	C_9_H_12_	1,2,4‐Trimethylbenzene
4	4.699	1120‐21‐4	C_11_H_24_	Undecane
5	4.787	2783‐26‐8	C_9_H_10_O	2‐(2‐Methylphenyl)oxirane
6	5.281	527‐84‐4	C_10_H_14_	1‐Isopropyl‐2‐methylbenzene
7	5.287	934‐74‐7	C_10_H_14_	1,3‐Dimethyl‐5‐ethylbenzene
8	6.993	627‐48‐5	C_3_H_5_NO	Ethyl cyanate
9	8.228	20307‐84‐0	C_15_H_24_	*δ*‐Elemene
10	9.586	87‐44‐5	C_15_H_24_	*β*‐Caryophyllene
11	9.804	18794‐84‐8	C_15_H_24_	(E)‐*β*‐Farnesene
12	10.075	89155‐85‐1	C_10_H_16_O	(2*E*,4*E*)‐3,7‐Dimethyl‐2,4,6‐octatrien‐1‐ol
13	10.086	6753‐98‐6	C_15_H_24_	*α*‐Caryophyllene
14	10.192	6829‐41‐0	C_7_H_11_NO_5_	Diethyl (hydroxyimino)malonate
15	10.292	17699‐05‐7	C_15_H_24_	*α*‐*trans*‐Bergamotene
16	10.463	23986‐74‐5	C_15_H_24_	Germacrene D
17	10.481	495‐61‐4	C_15_H_24_	*β*‐Bisabolene
18	10.692	1019577‐40‐2	C_8_H_11_NO_3_	Methyl N‐(2‐furylmethyl)glycinate
19	10.839	20307‐83‐9	C_15_H_24_	*β*‐Sesquiphellandrene
20	12.039	17202‐57‐2	C_9_H_14_O_2_	Ethyl spiro[2.3]hexane‐1‐carboxylic acid ethyl ester
21	12.616	1139‐30‐6	C_15_H_24_O	Caryophyllene Oxide
22	12.621	74744‐54‐0	C_16_H_28_	(*Z*)‐4‐Hexadecen‐6‐yne
23	12.951	19888‐34‐7	C_15_H_24_O	(−)‐Humulene epoxide II
24	13.233	26184‐88‐3	C_15_H_26_O_2_	Bisabolol oxide B
25	13.251	6750‐60‐3	C_15_H_24_O	Spathulenol
26	13.61	515‐69‐5	C_15_H_26_O	*α*‐Bisabolol
27	17.145	112‐39‐0	C_17_H_34_O_2_	Methyl palmitate
28	22.239	13481‐95‐3	C_19_H_36_O_2_	10‐Octadecenoic acid methyl ester

The peak area of more than half of the components of ESF in volatile and fatty oils accounted for more than 70% of the total peak area of each sample, indicating that the identified compounds could represent the main components of ESF in volatile and fatty oils (Tables 4 and 5). Moreover, α‐bisabolol accounted for most of the chemical components of ESF detected in volatile oil. In components of ESF in fatty oil, 10‐octadecenoic acid methyl ester accounted for the most of chemical components.

**TABLE 4:** Relative area percentage of common peaks in volatile ESF components from different regions (S1–S15).

Peak number	Relative area percentage (%)														
	S1	S2	S3	S4	S5	S6	S7	S8	S9	S10	S11	S12	S13	S14	S15
1	1.742	2.266	3.958***	0.708***	0.786***	1.878	3.616***	4.393***	0.437***	–***	–***	0.563***	4.501***	1.65	0.432***
2	–	–	0.543***	–	–	–	–	0.635***	–	–	–	–	–	–	–
3	0.943	–***	–***	–***	–***	0.495***	0.294***	–***	–***	0.49***	–***	–***	–***	0.442***	–***
4	0.471	0.708***	1.19***	0.433	0.538*	0.737***	0.957***	1.39***	–***	–***	–***	–***	1.216***	0.588**	2.232***
5	5.936	2.403***	3.857**	2.38***	3.808**	3.265***	2.534***	4.487**	2.324***	2.233***	2.031***	2.316***	1.348***	3.42**	2.232***
6	–	6.456***	8.917***	8.215***	–	7.036***	7.583***	7.237***	–	–	5.476***	6.583***	2.308***	8.466***	7.994***
7	–	–	–	–	–	–	0.981***	1.007***	–	–	–	1.287**	–	–	1.58***
8	1.223	0.543***	1.649**	1.071**	0.915***	1.244	–***	–***	1.815***	0.865***	–***	–***	–***	1.062**	–***
9	3.825	1.743***	3.14**	2.114**	3.216	2.671**	2.125***	2.951***	2.563**	1.92***	3.037	2.26**	0.697***	2.9	1.885***
10	0.581	–***	1.164***	0.695*	0.834***	0.687*	0.532	1.245*	0.611	0.638	–***	0.51	0.947**	0.68	–***
11	–	–	0.593***	–	–	–	–	0.277***	–	–	–	–	–	–	–
12	–	–	–	–	0.824***	–	3.12***	–	–	–	–	0.735***	0.859***	2.624***	0.627***
13	–	–	–	–	–	6.928***	4.343***	13.475***	–	–	–	–	–	4.558***	–
14	7.031	–***	–***	–***	–***	–***	–***	–***	–***	–***	–***	4.757***	–***	–***	–***
15	–	–	0.691***	0.487***	0.668***	0.544***	0.531***	1.409**	–	0.923***	–	–	1.38**	–***	1.867***
16	6.921	6.753	6.101	7.996	7.247	6.613	4.973***	5.997**	6.7	6.965	5.974**	6.58	3.674***	7.77	8.052**
17	–	–	0.245***	–	–	–	–	–	–	–	–	–	–	–	0.436***
18	1.388	5.521***	0.984	0.845	0.818	1.67*	2.022**	2.339*	0.738	0.987	–***	0.76	2.908***	2.587***	1.974**
19	–	–	–	–	0.497**	–	–	–	–	–	–	–	–	0.397**	–
20	–	–	0.462***	–	–	–	–	0.553***	–	–	–	–	0.528***	–	–
21	0.707	0.824	0.6	0.821	1.46**	0.836	0.523	1.506**	0.795	1.055	–***	0.72	1.688**	0.89	0.62
22	–	–	2.058***	–	–	–	2.186***	–	2.13***	2.079***	–	1.805***	–	–	–
23	–	1.197***	0.732***	–	–	0.799***	–	–	0.809***	–	–	–	–	1.01***	0.923***
24	–	–	–	–	–	–	0.507**	–	–	–	–	–	–	0.428**	–
25	–	–	0.421**	–	0.354**	–	–	0.315**	–	–	–	–	–	–	–
26	–	–	0.249***	–	0.517***	–	–	–	–	–	–	–	–	–	–
27	1.253	1.49	0.923	1.118	11.013***	1.475	0.997	1.61	1.494	3.051***	–***	1.6	2.29	1.59	0.8
28	1.037	0.91	1.951***	1.792***	7.905***	1.907***	1.718***	1.72***	2.237***	2.531**	–***	2.743**	0.87	2.185***	2.091***
29	–	1.225***	3.214***	2.524***	–	2.532***	2.265***	2.134***	2.216***	1.929***	–	2.416***	–	2.106***	1.893***
30	–	–	0.819***	0.998***	2.313***	1.126***	–	0.806***	–	–	–	–	–	–	–
31	–	–	–	–	–	–	0.755***	–	1.509**	1.198***	–	1.95***	–	–	0.882***
32	–	–	0.214**	–	0.29***	–	–	–	–	–	–	–	–	–	–
33	–	0.565*	0.776*	1.393***	–	–	–	–	–	–	–	1.109***	–	–	–
34	–	0.879***	–	1.278***	–	–	–	0.939***	–	–	–	–	–	–	1.456**
35	–	8.155***	–	–	–	–	–	–	–	10.743***	–	–	7.939***	–	6.162***
36	–	–	–	–	0.816***	–	–	–	–	–	–	–	–	0.449***	–
37	35.394	43.636***	22.151***	38.933***	22.667***	39.37***	25.706***	26.39***	42.828***	44.708***	–***	45.337***	43.903***	38.047***	42.508***
Total	68.452	85.274	67.602	73.801	67.486	81.813	68.268	82.818	69.206	82.315	16.518	84.022	77.055	83.846	86.643

*Note*: “–”indicates that the value is not detected or the relative content is too low. “*” indicates a statistical difference. “**” indicates a significant statistical difference. “***” indicates an extremely significant statistical difference.

**TABLE 5:** Relative area percentage of common peaks in fatty ESF components from different regions (S1–S15).

Peak number	Relative area percentage (%)														
	S1	S2	S3	S4	S5	S6	S7	S8	S9	S10	S11	S12	S13	S14	S15
1	0.664	0.879	0.536	0.441	–***	0.385	0.408	0.477	0.977	0.21	0.399	0.322	0.842	0.519	0.457
2	0.911	1.45	0.772	0.691	3.304***	0.545	0.615	0.722	0.302*	0.215*	0.626	0.594	1.383	0.858	0.16*
3	–	1.45***	0.198***	0.691***	–	–	–	–	–	–	–	–	–	–	–
4	0.611	–***	–***	–***	–***	–***	0.38	0.407	–***	–***	–***	–***	–***	–***	–***
5	–	–	–	–	0.523***	–	–	–	–	–	–	–	–	0.222***	–
6	–	–	–	–	0.4***	–	–	–	0.316***	–	–	–	–	–	–
7	–	–	0.158***	–	0.376***	–	–	–	–	–	–	–	–	–	–
8	–	–	–	–	–	–	–	–	–	0.251***	–	–	–	0.235***	–
9	–	1.087**	2.114***	–	–	2.673***	1.116***	–	–	–	12.057***	–	2.545***	0.781***	0.777***
10	–	–	–	–	–	1.479***	–	1.925***	–	–	–	–	–	1.34***	–
11	2.414	3.825**	4.495***	2.019	–***	2.421	3.66**	4.525***	2.478	2.153	–***	–***	–***	4.559***	–***
12	–	1.059**	–	0.415***	–	–	–	–	–	–	–	–	–	–	–
13	–	–	1.294***	–	–	0.975***	0.851***	1.415***	–	–	–	1.137***	–	1.135***	–
14	–	–	0.344*	–	–	–	–	–	–	–	0.505***	–	–	–	–
15	–	–	–	–	–	2.941***	0.977***	–	–	–	–	–	–	1.011***	–
16	–	–	1.351***	–	–	–	1.44***	–	–	–	4.037***	–	15.073***	2.024***	–
17	–	4.957***	3.562***	3.143***	–	4.136***	1.294***	3.644***	2.949***	3.209***	–	4.92***	–	4.789***	1.108***
18	–	1.292***	–	–	–	–	–	–	0.928***	–	–	0.177***	–	–	–
19	–	–	–	–	–	–	–	–	–	–	–	0.7***	–	1.104***	–
20	–	–	–	–	–	–	–	0.27***	–	–	–	–	–	–	0.468**
21	–	–	–	–	1.866***	0.555***	1.504***	1.537***	–	0.945***	–	1.281***	–	1.078***	–
22	–	–	1.51***	–	–	–	–	–	–	–	–	–	–	–	0.766***
23	–	–	–	–	0.616***	–	0.487***	–	–	–	–	–	–	–	–
24	–	–	0.619***	–	–	0.556***	0.79***	0.946***	1.08***	0.758***	–	–	–	1.159***	0.578***
25	3.65	9.582***	–***	4.673**	3.519	0.625***	8.776***	5.131**	5.088**	2.2386**	–***	3.348	–***	–***	–***
26	13.353	38.999***	12.177**	17.625***	2.312***	22.442***	18.76***	14.772**	20.847***	12.826	0.772***	17.142***	17.154***	21.402***	12.572*
27	1.364	–***	1.247	1.331	–***	0.855	1.603	1.625	0.941	0.886	3.7***	–***	0.831	–***	0.891
28	–	19.637***	–	62.544***	63.006***	35.697	–	49.818***	–	–	32.816***	55.99***	39.38***	–	69.534***
Total	22.967	84.217	30.377	93.573	75.922	76.285	42.661	87.214	35.906	23.6916	54.912	85.611	77.208	42.216	87.311

*Note*: “–” indicates that the relative content is not detected or is too low. “*” indicates a statistical difference. “**” indicates a significant statistical difference. “***” indicates a extremely significant statistical difference.

Relative area percentage of common peaks of the components of ESF in volatile oil showed that the contents of α‐bisabolol and β‐bisabolene were the highest in S12 and S15, which were 45.34% and 8.052%, respectively. In the components of ESF in fatty oil, 10‐octadecenoic acid methyl ester exhibited to have the highest content in S15 (69.53%) and S5 (63.01%). The results revealed that the main components of volatile substance were similar in different regions, while the content was different.

**FIGURE 2:**
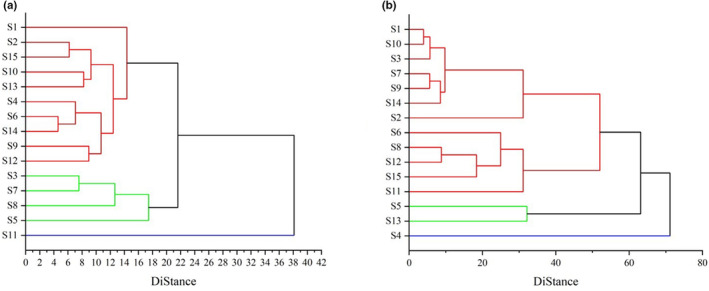
Cluster analysis of volatile ESF components (a) and fatty ESF components (b) from different regions (S1–S15).

It was indicated that S1, S2, S15, S10, S13, S4, S6, S14, S9, and S12 belonged to the same category, and S3, S7, S8, and S5 belong to the same category in volatile oil (Figure 2a). The distance between the two categories was only 2, confirming that the components of ESF in volatile oil from these two production areas were similar with a relatively satisfactory quality, and S11 belonged to the same category independently in volatile oil. Similarly, S1, S10, S3, S7, S9, S14, S2, S6, S8, S12, S15, and S11 belonged to the same category, and S5 and S13 belong to the same category in fatty oil (Figure 2b). The distance between these two categories was only 3, proving that the quality of the components of ESF in fatty oil from these two categories was relatively satisfactory. Furthermore, S4 belonged to the same category independently, and the distance between the other two categories was 40, indicating that the quality of the components of ESF in fatty oil in this area was relatively poor.

### 3.2 Nonvolatile components of ESF

**FIGURE 3:**
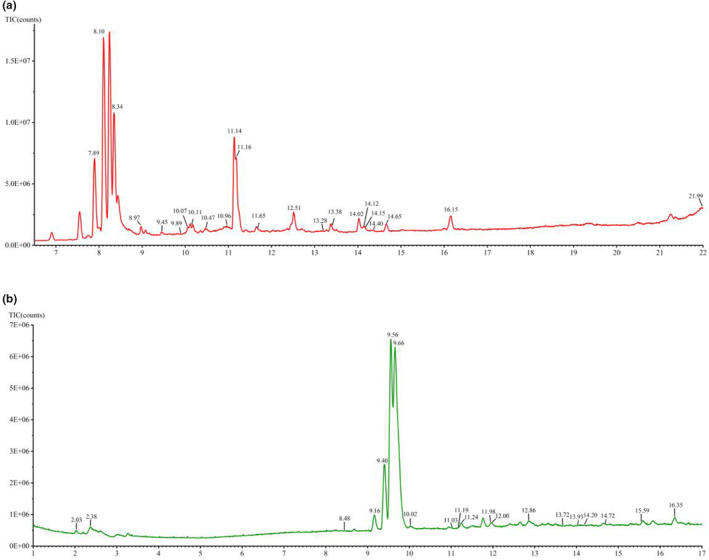
The BPI chromatograms of nonvolatile ESF components were detected at 7–22 min in positive ion mode (a) and at 1–17 min in negative ion mode (b).

According to the exact fragmentation rules of fragment ions and literature, 43 compounds were identified (Figure 3, Table 6), which were mainly triterpene and phenylpropanoid (Hu et al., 2022; Liu et al., 2021).

**TABLE 6:** Characterization of non–volatile ESF components by UPLC–MS/MS.

No.	Identification	*t*_R_ (min)	Characteristic fragment ions	*m*/*z*	Formula	Neutral mass
1	(+)‐Kobusin	2.03	341.10934 [M‐2CH_3_ + H]^−^, 323.09912 [M‐CH_3_‐CH_2_O‐2H]^−^	415.1407 [M + HCOO]^−^	C_21_H_22_O_6_	370.14164
2	Evernic Acid	2.38	300.26321 [M‐OCH_3_‐H]^−^	377.0868 [M + HCOO]^−^	C_17_H_16_O_7_	332.08960
3	Cussonoside A	7.89	665.37033 [M‐Rha‐CH_2_OH‐C_6_H_12_ + H]^+^	981.4849 [M + K]^+^	C_48_H_78_O_18_	942.51882
4	Quercetin	8.10	149.05449 [M‐C_7_H_6_O_4_ + H]^+^	303.0495 [M + H]^+^	C_15_H_10_O_7_	302.04265
5	n‐Butyl‐1‐O‐*α*‐l‐rhamnopyranoside	8.34	58.16252 [M‐Rha + H]^+^	221.1382 [M + H]^+^	C_10_H_20_O_5_	220.13107
6	Neochlorogenic acid	8.48	162.05479 [M‐C_7_H_11_O_6_‐H]^−^	353.0884 [M‐H]^−^	C_16_H_18_O_9_	354.09508
7	Betulonic acid	8.97	437.33864 [M‐H_2_O]^+^, 252.15799 [M‐C_11_H_18_O‐CH_3_‐H_2_O]^+^, 203.17790 [M‐CO_2_‐C_14_H_22_O]^+^	455.3519 [M + H]^+^	C_30_H_46_O_3_	454.34470
8	2,6‐Dimethoxy‐4‐(1*E*)‐3,3‐dimethoxy‐1‐propenyl]phenyl *β*‐d‐glucopyranoside	9.16	281.82769 [M‐Glc‐H]^−^	461.1671 [M + HCOO]^−^	C_19_H_28_O_10_	416.16825
9	Ecliptasaponin A	9.40	588.04579 [M‐CO_2_‐H]‐, 409.20641 [M‐CO_2_‐Glc‐H]^−^	679.4074 [M + HCOO]^−^	C_36_H_58_O_9_	634.40808
10	(4R,6R)carveol *β*‐d‐glucopyranoside	9.45	151.07346 [M‐C_3_H_5_‐C_4_H_8_O_4_‐2H]^+^	315.1799 [M + H]^+^	C_16_H_26_O_6_	314.17294
11	4‐Hydroxycinnamic acid	9.56	118.07346 [M‐CO_2_‐H]‐, 92.17790 [M‐C_2_H_2_‐CO_2_‐H]^−^	209.0453 [M + HCOO]^−^	C_9_H_8_O_3_	164.04734
12	(+)‐Pinoresinlo‐di‐O‐*β*‐d‐glucoside	9.66	357.13283 [M‐Glc + H]^−^	519.1850 [M‐H]^−^	C26H32O11	520.19446
13	Isorhamnetin‐3‐*O*‐glucoside	9.89	342.06377 [M‐OH‐C_4_H_8_O_4_ + H]^+^, 176.04955 [M‐Glc‐C_6_H_4_O_3_ + H]^+^	501.1015 [M + NA]^+^	C_22_H_22_O_12_	478.11113
14	Kaempferol 7‐*O*‐glucoside	10.02	366.06153 [M‐CH_2_OH‐_3_H_2_O]^−^	447.0914 [M‐H]^−^	C_21_H_20_O_11_	448.10056
15	Avicularin	10.07	384.09103 [M‐3H_2_O + H]^+^	457.0760 [M + NA]^+^	C_20_H_18_O_11_	434.08491
16	3‐*O*‐Arabinopyranosyloleanolic acid	10.11	544.17278 [M‐CO_2_ + H]^+^, 441.15342 [M‐Ara + H]^+^	627.3679 [M + K]^+^	C_35_H_56_O_7_	588.40260
17	Ferulic acid	10.47	163.03914 [M‐H_2_O‐CH_3_ + H]^+^, 135.04412 [M‐CHCOOH‐H]^+^, 133.02838 [M‐CO_2_‐CH_3_‐H]^+^	217.0474 [M + NA]^+^	C10H10O4	194.05791
18	Glycyrrhizic acid	10.96	691.36587 [M‐C_5_H_8_O_2_‐2H_2_O + 3H]^+^, 415.28821 [M‐C_4_H_7_‐C_12_H_17_O_12_ + H]^+^	845.3917 [M + NA]^+^	C42H62O16	822.40379
19	Ciwujianoside C3	11.03	908.35894 [M‐Ara‐H]^−^, 761.25119 [M‐Ara‐Rha‐H]^−^, 599.26944 [M‐Ara‐Rha‐Glc‐H]^−^	1103.5655 [M + HCOO]^−^	C_53_H_86_O_21_	1058.56616
20	Chikusetsu saponin IVa	11.14	439.35894 [M‐CH_3_‐C_7_H_11_O_7_‐C_6_H_9_O_6_]^+^	817.4324 [M + NA]^+^	C_42_H_66_O_14_	794.44526
21	3′‐Methoxydaidzin	11.16	429.11812 [M‐H_2_O]^+^, 385.09270 [M‐C_2_H_4_O_2_]^+^, 313.06892 [M‐C_5_H_10_O_4_ + H]^+^, 297.03718 [M‐C_5_H_10_O_4_‐CH_3_]^+^	469.1112 [M + NA]^+^	C_22_H_22_O_10_	446.12130
22	lariciresinol‐4’‐*O*‐*β*‐d‐glucoside	11.19	359.15553 [M‐Glc‐H]^−^, 329.14376 [M‐Glc‐OCH_3_‐H]^−^	521.2093 [M‐H]^−^	C_26_H_34_O_11_	522.22576
23	*p*‐Anisic acid	11.24	107.12763 [M‐CO_2_‐H]^−^, 75.08737 [M‐CO_2_‐OCH_3_‐H]^−^	151.0403 [M‐H]^−^	C_8_H_8_O_3_	152.04734
24	(+)‐Simplexoside	11.65	357.13201 [M‐Glc + H]^+^	541.1780 [M+ NA]+	C_26_H_30_O_11_	518.06847
25	4′‐Methoxypuerarin	11.98	414.16903 [M‐OCH_3_‐H]^−^, 251.02349 [M‐OCH_3_‐Glc‐H]^−^	445.1133 [M‐H]^−^	C_22_H_22_O_10_	446.12130
26	3′‐Methoxypuerarin	12.00	414.02815 [M‐OCH_3_‐H]^−^, 234.11197 [M‐Glc‐H_2_O‐H]^−^	491.1201 [M + HCOO]^−^	C_22_H_22_O_10_	446.12130
27	Tetracentronside B	12.51	359.14754 [M‐Glc + H]^+^, 323.13105 [M‐Glc‐ H_2_O + H]^+^	543.1832 [M+ NA]+	C_26_H_32_O_11_	520.04226
28	3‐O‐*α*‐Rhamnopyranosyl‐(1 → 2)‐*α*‐arabinopyranoside‐29‐hydroxy leanolic acid	12.86	571.03797 [M‐Rha‐CH_2_OH‐H]^−^, 423.08432 [M‐Rha‐Ara‐CH_2_OH‐H]^−^	749.4471 [M‐H]^−^	C_41_H_66_O_12_	750.45543
29	3‐Methoxy‐4‐hydroxycinnamyl *β*‐d‐glucopyranoside	13.28	272.11082 [M‐4H_2_O‐2H]^+^, 248.11351 [M‐2H_2_O‐C_2_H_4_O_2_]^+^	365.1191 [M + NA]^+^	C16H22O8	342.13147
30	Isolariciresinol‐4‐O‐β‐d‐glucopyranoside	13.38	219.1025 [M‐Glc‐C7H8O + Na]^+^	545.1993 [M+ NA]+	C_26_H_34_O_11_	522.03169
31	3‐O‐*α*‐Arabinopyranoside 29‐hydroxy oleanolic acid	13.72	439.11197 [M‐Ara‐CH_2_OH‐H]^−^	603.3906 [M‐H]^−^	C_35_H_56_O_8_	604.39752
32	3‐O‐*β*‐Glucopyranosyl‐(1 → 2)‐α‐arabinoside‐29‐hydroxy oleanolic acid	13.93	555.07846 [M‐Glc‐CH_2_OH‐H]^−^, 423.55875 [M‐Glc‐Ara‐CH_2_OH‐H]^−^	765.4399 [M‐H]^−^	C_41_H_66_O_13_	766.45034
33	Kaempferitrin	14.02	429.11796 [M‐2H_2_O‐C_5_H_10_O_3_ + 3H]^+^, 385.09203 [M‐CH_3_‐OH‐Rha + 2H]^+^, 341.06559 [M‐C_3_H_6_O_2_‐Rha]^+^, 237.07618 [M‐Rha‐2H_2_O + H]^+^	617.1257 [M + K]^+^	C_27_H_30_O_14_	578.16356
34	3‐Phenylpropionic acid	14.12	135.08050 [M‐H_2_O + 2H]^+^, 107.08552 [M‐CO_2_ + 2H]^+^	151.0756 [M + H]^+^	C_9_H_10_O_2_	150.06808
35	Coniferaldehyde glucoside	14.15	323.11595 [M‐H_2_O]^+^	341.1242 [M + H]^+^	C_16_H_20_O_8_	340.11582
36	Eclalbasaponin I	14.20	778.13573 [M‐H_2_O‐H]^−^	795.4522 [M‐H]^−^	C_42_H_68_O_14_	796.46091
37	Hederagenin 3‐O‐*β*‐d‐glucuronopyranosyl methyl ester‐28‐O‐*β*‐d‐glucopyranoside	14.40	542.32958 [M‐CH_2_OH‐C_10_H_16_O_7_‐3H]^+^, 344.31628 [M‐2C_7_H_11_O_7_‐C_5_H_10_ + 4H]^+^	847.4426 [M + NA]^+^	C_43_H_68_O_15_	824.45582
38	(7*S*,8*R*)‐dihydrodehyd‐rodiconiferyl alcohol‐4‐O‐*β*‐d‐glucopyranoside ((7*S*,8*R*)‐urolignoside	14.65	371.17862 [M‐3OCH_3_‐CH_6_OH + H]^+^, 192.111595 [M‐3OCH_3_‐CH_6_OH‐Glc + H]^+^	561.1734 [M + K]^+^	C_26_H_34_O_11_	522.21011
39	Silphioside F	14.72	393.14402 [M‐2CO_2_‐Xyl‐H]^−^	631.3879 [M‐H]^−^	C_36_H_56_O_9_	632.39243
40	*α*‐Hederin	15.59	455.36219 [M‐Ara‐Rha]^−^	733.4557 [M‐H]^−^	C_41_H_66_O_11_	734.46051
41	Glycosides E1	16.15	439.35516 [M‐Glc‐Ara‐H_2_O + H]^+^, 351.0621 [M‐HCOOH+H]^+^	773.4457 [M + NA]^+^	C_41_H_66_O_12_	750.50317
42	Wujiapioside B	16.35	617.06817 [M‐Rha‐Glc‐H]^−^	987.5156 [M + HCOO]^−^	C_48_H_78_O_18_	942.51882
43	(−)‐Schisandrin B	21.99	237.15238 [M‐C_9_H_8_O_3_ + H]^+^	401.1942 [M + H]^+^	C_23_H_28_O_6_	400.18859

### 3.3 Analysis of triterpenoids

**FIGURE 4:**
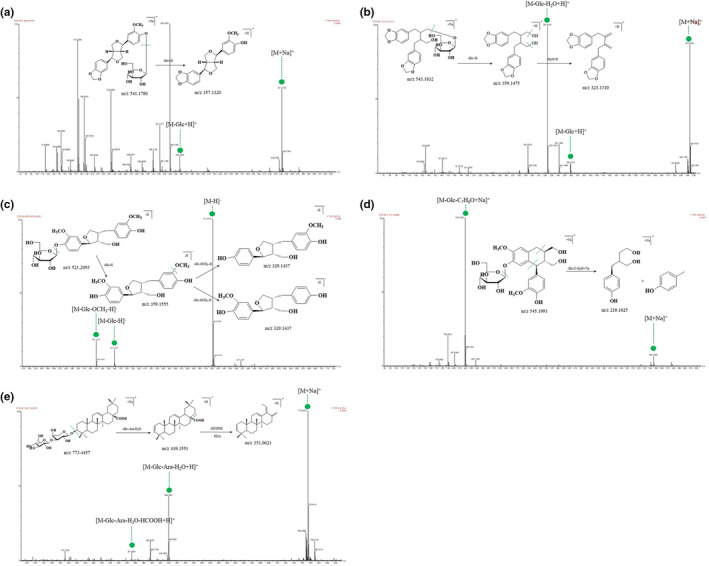
The cleavage pathways of (+)‐simplexoside (a), tetracentronside B (b), lariciresinol‐4’‐*O*‐*β*‐d‐glucoside (c), isolariciresinol‐4‐*O*‐*β*‐d‐glucopyranoside (d), and glycosides E1(e).

To date, no systematic characterization of triterpenoid in ESF by UPLC–MS/MS has been reported. A total of 16 triterpenoid saponins have been identified in this study. According to their structural characteristics, they were mainly oleanolic acid type. In the positive and negative ion modes, the additional ions of triterpenoid saponin were mainly [M + Na]^+^, [M + H]^+^, [M‐H]^−^, and [M + HCOO]^−^, and the nuclear parent fragment was obtained by breaking or continuously breaking *O*‐glycosyl or glycosyl. It included glucose (162 Da), rhamnose (146 Da), glucuronic acid (176 Da), galactose (162 Da), xylose (132 Da), and arabinose (132 Da). The possible cleavage pathway of triterpenoids was deduced in the positive ion mode as represented by compound 41. The quasi‐molecular ion peak of [M + Na]^+^ was *m*/*z* 733.4457 (C_41_H_66_O_12_). First, one glucose fragment ion, one arabinose fragment ion, and one neutral fragment H_2_O were removed to obtain the *m*/*z* 439.3551 (C_30_H_46_O_2_) fragment. The aglycone was further fragmented by the retro‐Diels–Alder (RDA) fragmentation. Due to the presence of carboxyl, it was easy to lose HCOOH fragment and obtain *m*/*z* 351.0621 (C_26_H_38_) fragment. Therefore, compound 41 was preliminarily identified as glycosides E1, and the cleavage pathway is shown in Figure 4e.

### 3.4 Analysis of phenylpropanoids

Six of the nine phenylpropanoid compounds identified were lignans with the same parent nucleus. These lignans generally break CO (28 Da), OCH_3_ (31 Da), and some glycosyl. In addition, the 7 and 7′ positions of the benzene ring are prone to fracture, resulting in benzyl cleavage. If there is hydroxyl group on the side chain benzene ring, it can form OCH_2_O (46 Da) characteristic fragments with the methoxy group. With compound 24 as the representative in the positive ion mode, the possible cleavage pathway of the bisepoxylignans was speculated. The quasi‐molecular ion peak of [M + Na]^+^ was *m*/*z* 541.1780 (C_26_H_30_O_11_), and one Glc was lost. The fragment ion *m*/*z* 357.1320 (C_20_H_20_O_6_) was achieved. Therefore, compound 24 was preliminarily identified as (+)‐simplexoside, and the cleavage pathway is illustrated in Figure 4a.

Taking compound 22 in the negative ion mode as the example, the possible cleavage pathway of monoepoxylignans was speculated. The quasi‐molecular ion peak of [M‐H]^−^ was *m*/*z* 521.2093 (C_26_H_34_O_11_), and one Glc was lost to obtain the fragment ion *m*/*z* 359.1555 (C_20_H_24_O_6_). Losing another OCH_3_ was resulted in the fragment ion *m*/*z* 329.1437 (C_19_H_22_O_5_), with two possible fragments. Therefore, compound 22 was preliminarily identified as lariciresinol‐4′‐*O‐β*‐d‐glucoside, and the cleavage pathway is displayed in Figure 4b.

The possible cleavage pathway of simple lignans was deduced by compound 27. The quasi‐molecular ion peak of [M + Na]^+^ was *m*/*z* 543.1832 (C_26_H_32_O_11_), and one Glc was lost. The fragment ion *m*/*z* 359.1475 (C_20_H_22_O_6_) and the loss of two neutral fragments H_2_O led to achieve the fragment *m*/*z* 323.1310 (C_20_H_18_O_4_). Therefore, compound 27 was preliminarily identified as tetracentronside B, and the cleavage pathway is shown in Figure 4c.

In the positive ion mode, compound 30 was represented, and the possible cleavage pathway of cyclolignans was predicted. The quasi‐molecular ion peak of [M + Na]^+^ was *m*/*z* 545.1993 (C_26_H_34_O_11_), and one Glc was lost. Fracture occurs at the 7 and 7′ positions and the loss of C_7_H_8_O results in the fragment ion *m*/*z* 219.1025 (C_11_H_16_O_3_). Therefore, compound 30 was preliminarily identified as isolariciresinol‐4‐*O‐β*‐d‐glucopyranoside, and the cleavage pathway is illustrated in Figure 4d.

### 3.5 Determination of antioxidant activity

Several studies have characterized the nonvolatile, volatile, and fatty oil components in plants by GC–MS and UPLC–MS/MS, accompanied by antioxidant activities of components in plants (Ali et al., 2022; Al‐Nemari et al., 2020; Castillo et al., 2023; Duan et al., 2022; Hefny Gad et al., 2021). A previous study demonstrated that phenolic acids, represented by chlorogenic acid and caffeic acid, are the main reason for the antioxidant effect of ESF (Kim et al., 2015). However, the antioxidant effects of triterpene and phenylpropanoid in the nonvolatile components of ESF and the volatile and fatty oil components of ESF have not yet been studied.

**FIGURE 5:**
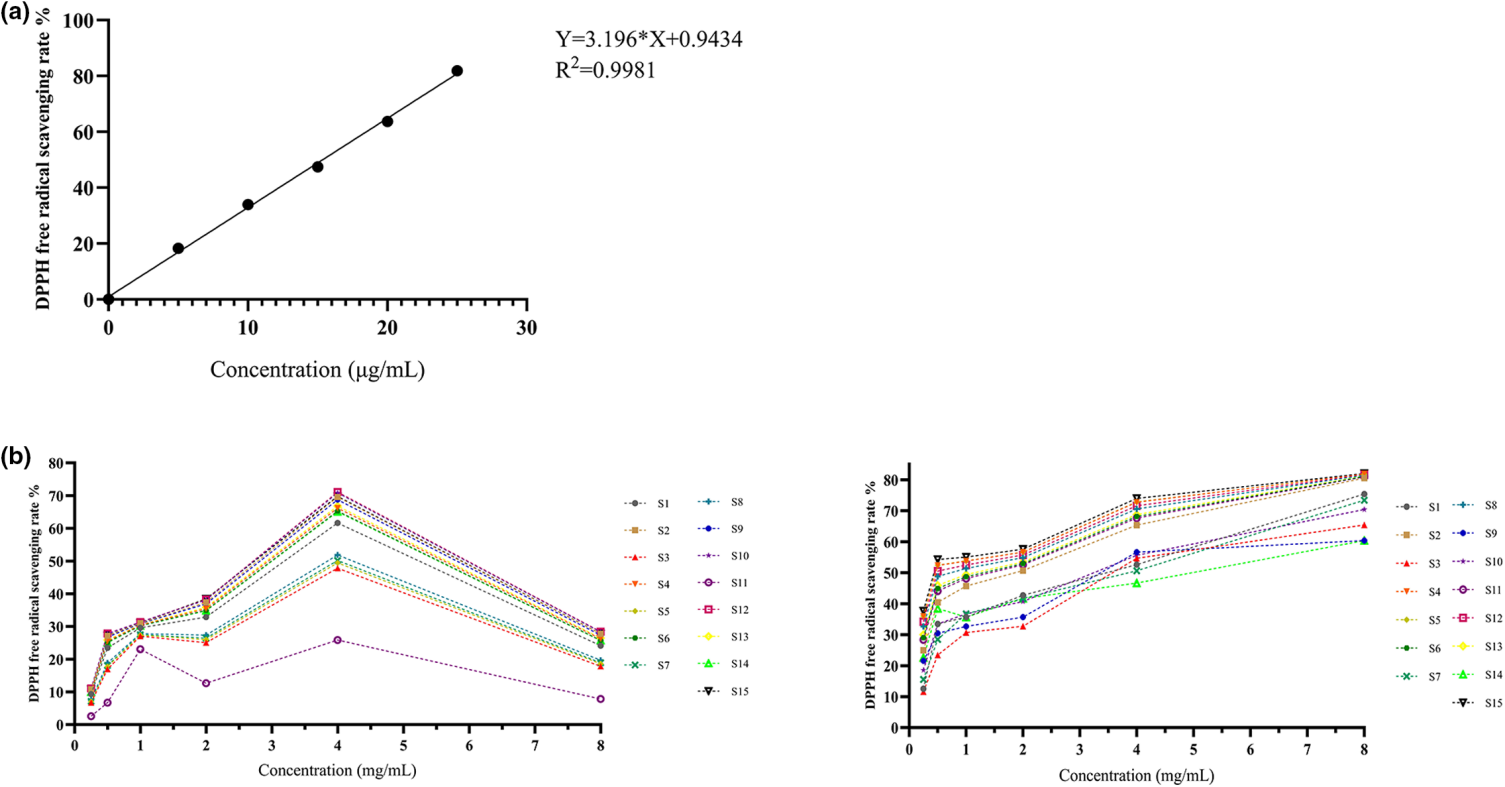
(a) DPPH radical scavenging standard curve. (b) Scavenging effects of volatile oil and fatty oil with different concentrations on DPPH free radical (S1–S15).

The changes of 2,2‐diphenyl‐1‐picrylhydrazyl (DPPH) radical scavenging abilities of the volatile and fatty oil of ESF with the concentration are shown in Figure 5. The scavenging ability of fatty oil on DPPH free radical was significantly stronger than that of volatile oil, and with the increase of concentration, the scavenging ability of fatty oil from different origins on DPPH free radical was gradually enhanced. S1, S7, S10, S3, S9, and S14 exhibited to have weaker than other production areas, and S15 had the strongest removal capacity. When the concentration of fatty oil in S15 reached 8.031 mg/mL, the scavenging rate was 82.04%, while the largest component in S15 was 10‐octadecenoic acid methyl ester. Moreover, this ingredient has been confirmed to have antioxidant activity, and it was speculated that this ingredient might have a certain relationship with the antioxidant activities of the components of ESF in fatty oil. The free radical scavenging abilities of the components of ESF in volatile oil from different production areas were not the same. Furthermore, S8, S7, S5, S3, and S11 also increased with the elevation of concentration before reaching 1.982 mg/mL, which did not show regularity. The scavenging abilities of the components of ESF in volatile oil from other production areas were enhanced with the increase of volatile oil concentration before reaching 4.022 mg/mL, and weakened to varying degrees when the concentration was greater than 4.022 mg/mL. Besides, S12 had the strongest scavenging ability, and the scavenging rate was 71.10% when the concentration of volatile oil reached 4.022 mg/mL. α‐Bisabolol, which accounted for the largest proportion in S12, has been confirmed to have antioxidant activity, and it was speculated that this ingredient might have a certain relationship with the antioxidant activity of the components of ESF in volatile oil.

**FIGURE 6:**
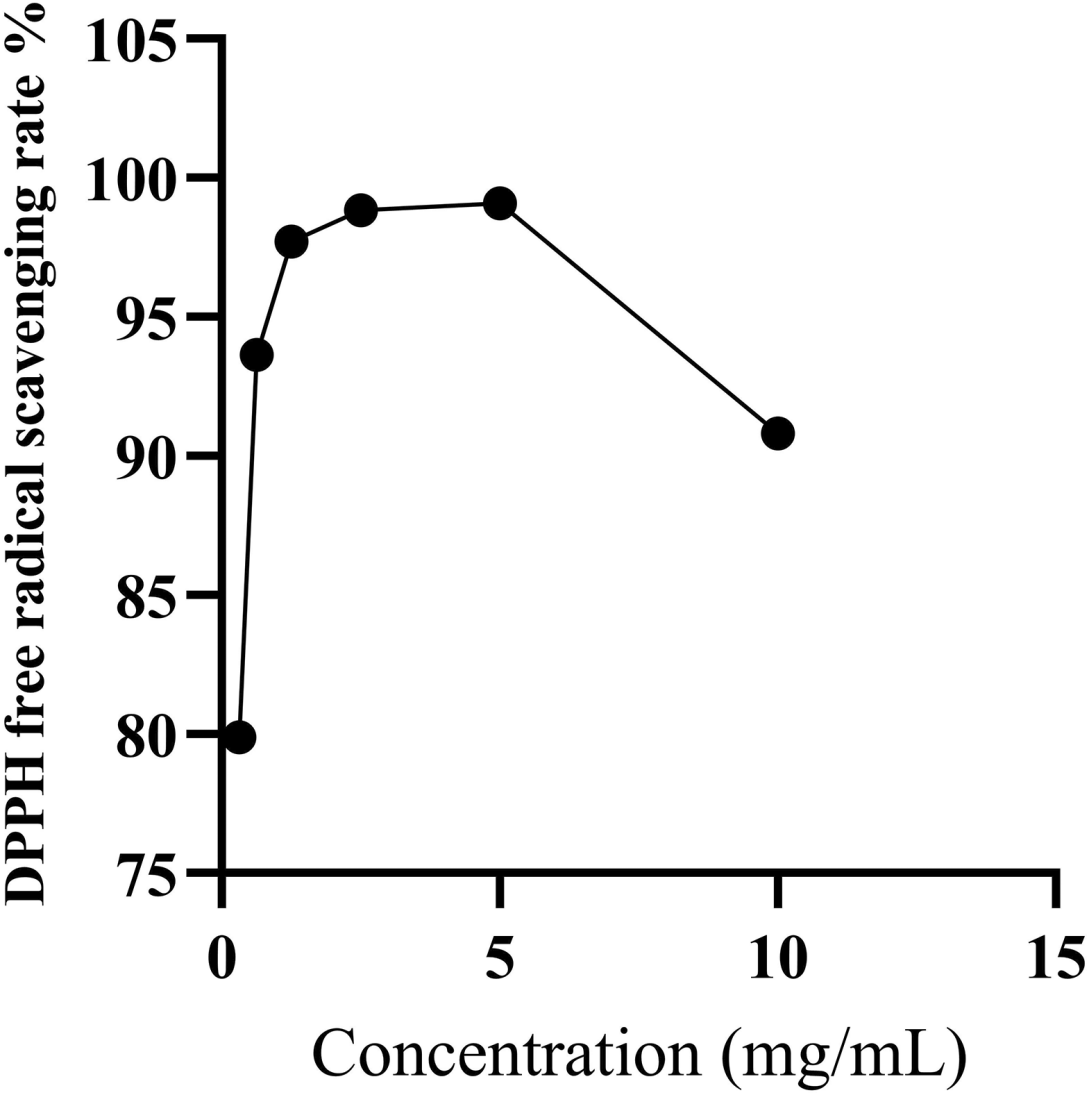
Scavenging effects of nonvolatile components with different concentrations on DPPH free radical.

The changes of DPPH radical scavenging abilities of nonvolatile components of ESF with the concentration are shown in Figure 6. When the concentration of sample was lower than 5.001 g/L, the free radical scavenging effect was gradually enhanced with the increase of concentration. When the concentration reached 5.001 g/L, the scavenging rate was about 99.09%, and then, with the elevation of the concentration, the scavenging effect on free radical decreased. The results of DPPH scavenging activity showed that nonvolatile components of ESF had strong scavenging ability on DPPH free radical, and their antioxidant activity was stronger than that of the volatile and fatty oil.

The studies have shown that sleep deprivation causes excessive oxidation of free radicals in brain tissue, leading to degenerative changes in tissues and cells, making excessive lipofuscin and malondialdehyde (MDA), thus damaging brain function and reducing learning and memory capacity. In this study, ESF has been proved to significantly improve the above symptoms and counter the effects of chronic sleep deprivation on the central nervous system (Zhang & Zhu, 2022). The main components of ESF volatile oil and fatty oil were terpene and fatty acid compounds by GC–MS analysis.

There is increasing evidence that terpene and fatty acid compounds have antioxidant activity (Wang et al., 2019). β‐Caryophllene alcohol (BCP), for example, inhibits oxidative stress and inflammation, reduce the activity of key enzyme myeloperoxidase (MPO) and the levels of key oxidative inflammatory factors reactive oxygen species (ROS) and oxidized glutathione (GSSG) in mouse liver (Ames‐Sibin et al., 2018), reduce neuropathic pain, prevent the increase of the content of malondialdehyde, the end product of lipid peroxidation, and thus improve the antioxidant capacity of cells (Aguilar‐Ávila et al., 2019). By activating transient receptor potential (TRP)V1, geranylacetone (GAT) makes Ca^2+^ flow in HEK293 cells and regulates the production of human neutrophils, thus producing antioxidant effects (Schepetkin et al., 2016).

Many fatty acid compounds can be obtained from food and used as a natural antioxidant, such as palmitic acid methyl ester (PAME), which dilates blood vessels and plays a significant role in brain damage caused by asphyxia cardiac arrest, elevated cholesterol, and cancer (Ichihara, 2021; Lee et al., 2019). Arachidic acid methyl ester plays a significant role for the prevention of gallstones by acting as a cholesterol solvent (Gilat et al., 2001). Methyl linoleate serves as an emulsifier in cosmetics and plays a direct role in the epidermal osmotic barrier, thus achieving an antioxidant effect (Qin et al., 2007).

In our study, ESF volatile oil and fatty oil have antioxidant effects, and its main components are α‐bisabolol and 10‐octadecenoic acid methyl ester. α‐Bisabolol was found to slow ROS production and inhibit the deposition of beta‐amyloid protein (Aβ) peptide induced by Alzheimer's disease in *Candida albicans* and *N*‐formyl‐methionyl‐leucyl‐phenylalanine(fMLP). Restoration of mitochondrial membrane potential (MMP) leads to antioxidant effects (Braga et al., 2009; Eddin et al., 2022; Gger et al., 2018). 10‐Octadecenoic acid methyl ester has been shown to lower blood cholesterol, have antifungal properties, and antioxidant effects (Belakhdar et al., 2015; Kewlani et al., 2022). However, there are few researches on the specific mechanism of its antioxidant. The study on the antioxidant activity of ESF volatile oil and fatty oil with terpene compounds and fatty acid compounds as the main components can be used as a new direction of ESF as a natural antioxidant in the food industry for the preparation of different health products. Moderate development of ESF can also provide another idea for the waste caused by excessive exploitation of ES roots and rhizome.

## 4 CONCLUSIONS

In this study, rapid and sensitive UPLC–QTOF–MS/MS plus GC–MS methods were developed for the analysis of nonvolatile and volatile components of ESF. Based on NIST14.L mass spectrometry database and precise molecular weight, 37 and 28 compounds were identified and analyzed from volatile oil and fatty oil of ESF, respectively, from different regions. The cluster analysis results of volatile oil showed that the distance between S11 and the other two categories was 16, and the cluster analysis results of fatty oil showed that the distance between S4 and the other two categories was 40, and the quality of oil from these two regions was significantly different from that of other producing areas.

In addition, 43 compounds were identified and analyzed from the nonvolatile components of ESF, and the cracking principles of some identified compounds were studied. DPPH antioxidant assay further verified that nonvolatile and volatile components of ESF might be associated with antioxidant activity. It has been suggested that ESF could be developed as a natural and potentially effective drug or functional food, however, its pharmacological action and related mechanisms need additional in vivo studies.

## AUTHOR CONTRIBUTIONS

**Yaodan Chang:** Conceptualization (equal); data curation (equal); resources (equal); software (equal); validation (equal); writing – original draft (equal). **Yong Jiang:** Data curation (equal); methodology (equal); visualization (equal). **Jingnan Chen:** Data curation (supporting); investigation (supporting). **Sen Li:** Data curation (supporting); formal analysis (supporting). **Yimeng Wang:** Visualization (supporting). **Linlin Chai:** Visualization (equal). **Jingwen Ma:** Formal analysis (supporting); visualization (supporting). **Zhibin Wang:** Funding acquisition (equal); project administration (equal); supervision (equal); validation (equal); writing – review and editing (equal).

## CONFLICT OF INTEREST STATEMENT

The authors declare that they do not have any conflict of interest.

## ETHICS STATEMENT

This study does not involve any human or animal testing.

## Supporting information


Figure S1

## Data Availability

The data that support the findings of this study are openly available in Food Science & Nutrition at 10.1002/fsn3.3887, reference number FSN3_3887.
