# Supplementary figures and images for: Comprehensive analysis of Eleutherococcus senticosus (Rupr. & Maxim.) Maxim. fruits based on UPLC–MS/MS and GC–MS: A rapid qualitative analysis
Source: Food Sci Nutr. 2023 Dec 13;12(3):1911–27. doi: 10.1002/fsn3.3887 (PMC10916571; doi:10.1002/fsn3.3887)

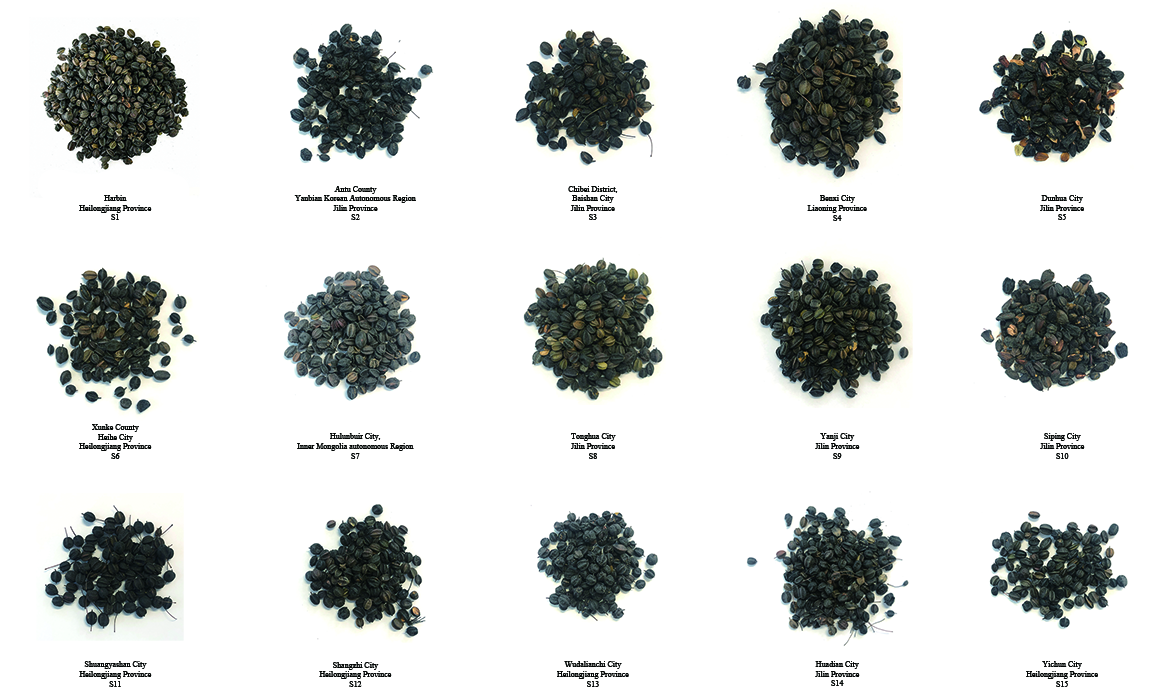

Supplement: Supplementary file 1 — Figure S1 [file FSN3-12-1911-s001.zip › fsn33887-sup-0001-FigureS1.tif]
